# SGLT2 Inhibition in HFpEF. Do We Need More Quantitative and Load Independent Metrics to Understand the Results of the EMPEROR-Preserved Trial?

**DOI:** 10.3389/fcvm.2021.822968

**Published:** 2022-01-14

**Authors:** Grigorios Korosoglou, Sorin Giusca, Sebastian Kelle

**Affiliations:** ^1^Department of Cardiology, Vascular Medicine and Pneumology, Gesundheitszentrum Rhein-Neckar Hospital Weinheim, Weinheim, Germany; ^2^Cardiac Imaging Center Weinheim, Hector Foundation, Weinheim, Germany; ^3^Department of Internal Medicine/Cardiology, Deutsches Herzzentrum Berlin, Berlin, Germany

**Keywords:** heart failure, preserved ejection fraction, SGLT2 inhibition, myocardial strain, fast-SENC

## Introduction

Despite advances in the pharmacologic and interventional treatment of ischemic, myocardial, and valvular heart diseases, heart failure is estimated to affect ~60 million individuals worldwide being associated with high morbidity and mortality rates. In a recent report by Anker and colleagues published in the New England Journal of Medicine, the authors demonstrated that SGLT2 inhibition with empagliflozin leads to relevant clinical outcome improvements, by reducing the relative risk for cardiovascular death and hospitalization for patients with symptomatic heart failure NYHA II-IV and preserved ejection fraction (HFpEF). The results were mainly driven by a reduction in hospitalization rates (1). We congratulate the authors for this article, which is to our knowledge the first randomized study, highlighting the ability of SGLT2 inhibition to improve clinical outcomes in HFpEF. Based on prespecified left ventricular ejection fraction (LVEF) subgroups, however, patients with LVEF ~40–49% mostly benefited from treatment, whereas positive effects were attenuated in patients with LVEF between 50 and 59% and were not statistically significant with LVEF ≥ 60%.

## Previous Studies with SGLT2 Inhibitors and Underlying Mechanisms

Several studies demonstrated the ability of SGLT2 inhibition to reduce cardiovascular endpoints in patients with heart failure and reduced ejection fraction regardless of the presence or absence of type 2 diabetes mellitus (2, 3). Hereby, the exact mechanism of action is still a subject of ongoing research. It has been previously proposed, that SGLT2 inhibition may exert beneficial effects by reducing inflammation, oxidative stress, and blood pressure due to diuresis and natriuresis, resulting in improvement of vascular and kidney function. In addition, beneficial effects in terms of cardiac energy metabolism have been described. Thus, SGLT2 inhibition may improve cardiac energetics and cardiac efficiency, by increasing circulating ketone levels and cardiac ketone oxidation rates, which can act as a thrifty fuel for the undersupplied “starving” failing heart (4). This may improve energy supply of the heart muscle, translating into lower rates of hospitalization due to heart failure symptoms, thus improving clinical outcomes.

## Technical Considerations and Discussion

**Figure 1:**
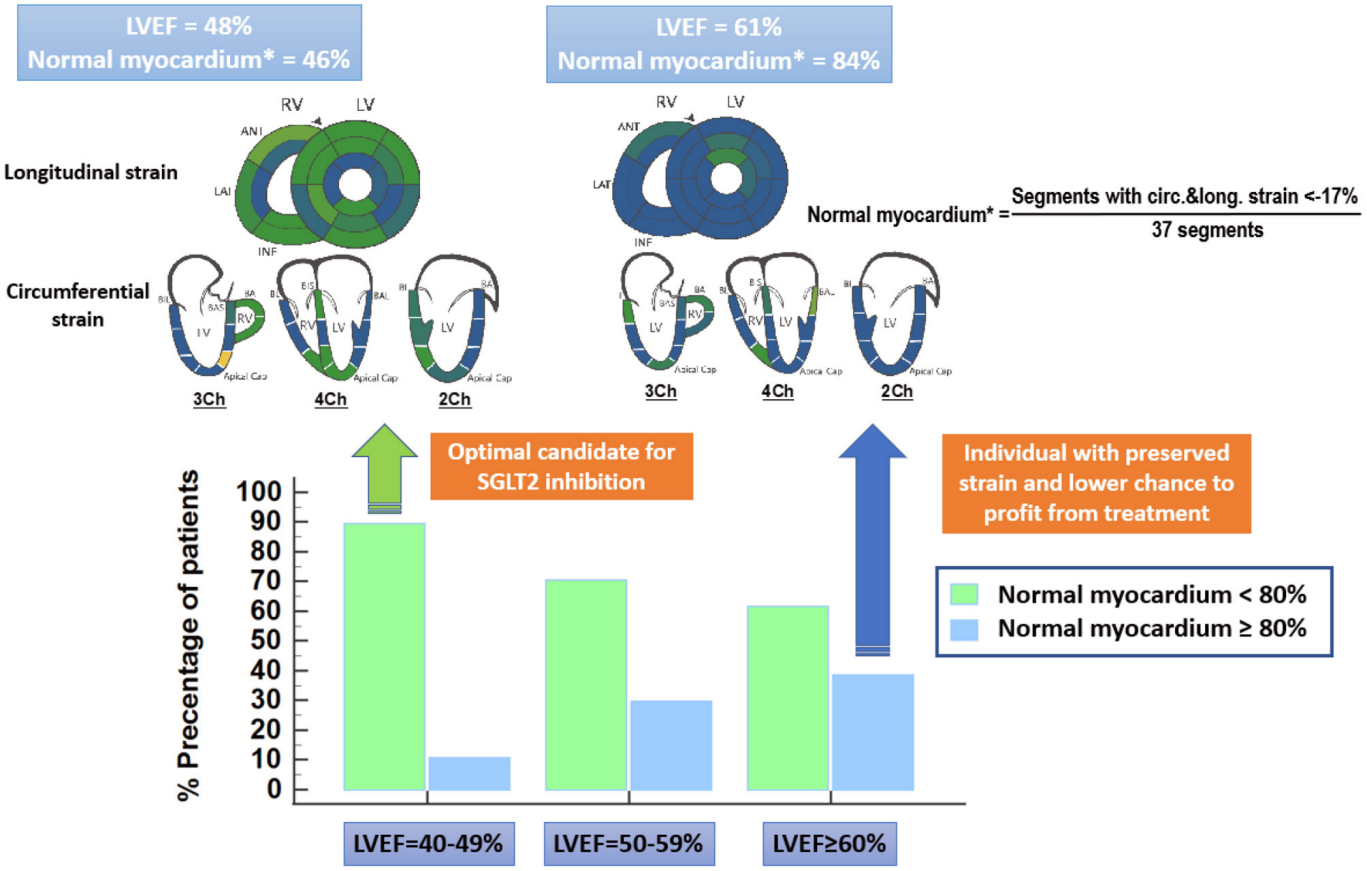
“Normal” myocardium is defined as the percentage of myocardial segments, which exhibit strain values ≤ −17% by advanced quantitative CMR using Fast Strain-encoded Cardiac Magnetic Resonance (fast-SENC). “Normal myocardium” < 80% is present in 89% patients with LVEF~40–49%, which are probably excellent candidates for SGLT2 inhibition (green arrow). Only 61% of patients with LVEF ≥ 60%, on the other hand exhibit “normal myocardium” < 80%. Patients which LVEF ≥ 60% and “normal myocardium” > 80% have rather another underlying disease mimicking heart failure and will not necessarily profit from SGLT2 inhibition (blue arrow).

From a pathophysiologic point of view, LVEF is a crude and load-dependent marker for cardiac efficiency (5, 6), whereas myocardial strain by echocardiography and advanced quantitative CMR, recently exhibited important value for the non-invasive assessment of myocardial fibrosis and incremental prognostic value beyond LVEF in heart failure patients (6–8). Thus, global longitudinal strain (GLS) was associated with clinical heart failure status, the level of neurohormonal activation and with long-term cardiac mortality in patients with asymptomatic and symptomatic heart failure (6). In addition, the presence of “normal” myocardium (defined as percentage of myocardial segments exhibiting a strain value ≤ −17%) by advanced quantitative CMR using Fast Strain-encoded Cardiac Magnetic Resonance (fast-SENC), recently demonstrated incremental value for the prediction of clinical outcomes beyond LVEF in an all-comer cohort of heart failure patients (7). In this study, more than one third of individuals who were classified just at risk for heart failure by conventional imaging markers including LVEF, were reclassified to patients with subclinical LV-dysfunction, exhibiting “normal” myocardium <80% (7). Such individuals with “normal” myocardium <80%, who were in most cases asymptomatic at baseline, showed higher rates for all-cause death and hospitalization due heart failure and for new onset of heart failure medications during follow-up (7). Since “normal myocardium” may represent a more valid surrogate marker of impaired myocardial energetics compared to LVEF, it is conceivable that patients with “normal myocardium” <80% are likely to exhibit unfavorable myocardial energetics and benefit from SGTL2 inhibition. In fact, impaired “normal myocardium” <80% was present in 89% patients with LVEF ~40–49% in our recent study, which are probably excellent candidates for SGLT2 inhibition but only in 61% patients with LVEF ≥ 60%, where SGLT2 inhibition may not necessarily translate to clinical benefits (Figure 1). Although GLS and normal myocardium have not been systematically analyzed in the EMPEROR preserved trial, these load independent metrics may aid in a more precise identification of appropriate candidates for SGLT2 inhibition with preserved LVEF in future trials.

In the same direction, CMR T1 mapping techniques allow for the assessment of interstitial space characteristics and extracellular volume size, which are related to collagen content and interstitial infiltration of myocardial tissue by fibrotic tissue or other molecules, such as amyloid. The ability of such measures for the risk stratification of patients with heart failure, cardiomyopathies or amyloidosis has already been demonstrated (8–11). In addition, recent studies highlighted the ability of such techniques to accurately assess longitudinal changes of myocardial extracellular volume in patients treated with SGLT2 inhibitors (12, 13).

Importantly, metrics such as strain or T1 values can be acquired serially during non-contrast CMR scans, thus without the need for gadolinium administration and without radiation exposure for the patients. Thus, such a direct measure of treatment response in terms of increases in strain and “normal myocardium” would be feasible in patients receiving SGLT2 inhibitors possibly a couple of weeks after the treatment initiation. Due to the quantitative nature of these parameters smaller populations than the one presented in the EMPEROR preserved trial would be necessary to investigate the direct effects of such drugs on myocardial strain in heart failure patients. Such advanced metrics like “normal myocardium” would therefore decrease trials costs and speed-up transfer of knowledge into clinical use, aiding individualized treatment of heart failure patients or even of asymptomatic individuals with subclinical LV-dysfunction.

## Author Contributions

All authors listed have made a substantial, direct, and intellectual contribution to the work and approved it for publication.

## Conflict of Interest

SK and GK received research grants from Myocardial Solutions. SK owns stock options of Myocardial Solutions. The remaining author declares that the research was conducted in the absence of any commercial or financial relationships that could be construed as a potential conflict of interest.

## Publisher's Note

All claims expressed in this article are solely those of the authors and do not necessarily represent those of their affiliated organizations, or those of the publisher, the editors and the reviewers. Any product that may be evaluated in this article, or claim that may be made by its manufacturer, is not guaranteed or endorsed by the publisher.
